# Diversifying Resistance Mechanisms in Cereal Crops Using Microphenomics

**DOI:** 10.34133/plantphenomics.0023

**Published:** 2023-02-13

**Authors:** Peter M. Dracatos, Stefanie Lück, Dimitar K. Douchkov

**Affiliations:** ^1^ Department of Animal, Plant and Soil Sciences, AgriBio, La Trobe University, Bundoora, VIC 3086, Australia.; ^2^ Leibniz Institute of Plant Genetics and Crop Plant Research (IPK) Gatersleben, Corrensstrasse 3, 06466 Seeland OT Gatersleben, Germany.

Whole-grain cereals, including wheat, barley, oat, rye, corn, rice, millet, and triticale, are a rich source of calories, essential vitamins, minerals, and phytochemicals that both nourish and protect humans and animals from diseases such as heart attack and cancer [1]. However, susceptibility to foliar diseases caused by necrotrophic or biotrophic fungal pathogens continues to reduce yield potential or lead to total crop failure and famine in developing nations [2]. Historically, foliar diseases of cereals have been controlled using either fungicide treatment [3] or plant breeding [4]. However, fungicides create selection pressure in favor of the emergence of insensitive pathogen variants and are both expensive and harmful to human health and the environment. In contrast, deploying disease resistance genes in improved cereal varieties has proven the most economical and environmentally sustainable approach to protect yield potential and ensure adequate quantities of pesticide-free food [5].

**Fig. 1.**
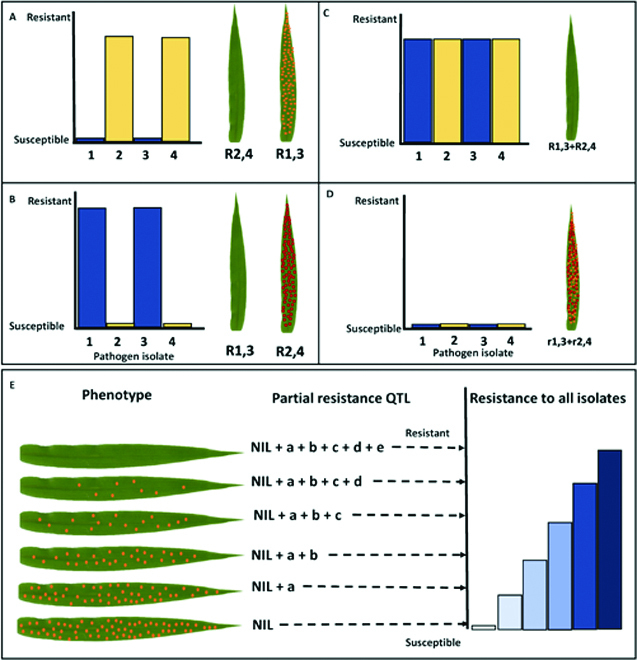
(A to D) For qualitative resistance, a single underlying gene will give rise to 2 distinct phenotypes: resistance or susceptibility. The resistance conferred by R1,3 and R2,4 is vertical and complete. The diagram of the race-specific qualitative phenotypic response of 4 cultivars carrying resistance genes (A) R1,3, (B) R2,4, (C) both R1,3 and R2,4, and (D) no R genes. Resistance gene R1,3 is effective only to pathogen races 1 and 3 (highlighted in blue), whereas R2,4 is effective to races 2 and 4 (highlighted in yellow). In combination, due to their race specificity, R1,3 and R2,4 confer enhanced resistance to races 1 to 4; however, they may not be effective to other untested pathogen races. The fourth cultivar is susceptible and carries no R genes or partial resistance. (E) For quantitative or horizontal resistance, there is no variation in phenotypic response to the different pathogen isolates. The reduction of infection sites observed for the phenotypic response of different host genotypes in a segregating near isogenic lines (NIL) population reflects the accumulation of distinct partial resistance quantitative trait loci (QTL) ranging from complete susceptibility (no QTL) to full immunity due to the presence of 5 QTLs conferring partial resistance. The segregation of quantitative resistance genes and environmental effects causes a range of resistance phenotypes.

Plant genotypes or populations exposed to pathogenic microbes often vary in their phenotypic response or degree of infestation, and this is due to differences in inherited defenses. Plant breeders and geneticists are constantly assessing visual symptoms of disease resistance traits in their experimental material. Although these data are valuable, its reproducibility is limited due to individual scoring biases, non-quantitative inoculation methods, and environmental variables. Currently, there is an imbalance between our ability to manipulate plant genomes and our capacity to phenotype accurately for disease, creating a bottleneck in the plant breeding pipeline. Despite next-generation sequencing reducing genotyping costs by more than 100-fold in the last 20 years, the cost and inaccuracy of disease phenotyping have impeded genetic gain. Furthermore, the requirement of phenotypic accuracy for trait dissection and breeding has led to a historic selection bias towards race-specific resistance genes of major phenotypic effect that are easier to phenotype but rarely durable in agricultural settings when deployed singly, due to rapidly evolving pathogen populations [6] (Fig. 1A to D).

In contrast, partial resistance (PR) refers to a reduction or delayed growth of the pathogen and is typically conditioned by several quantitatively inherited alleles (Fig. 1E). This form of resistance is durable, non-race-specific, and incomplete when considering the effect of a single locus in isolation [7]. However, the cumulative effect of several partial minor resistance loci often leads to complete immunity and has been reported to account for reduced disease epidemics. Both gene isolation and subsequent functional characterization studies in cereal crop plants demonstrate that PR genes show higher mechanistic diversification relative to the R genes explaining their durability [8–10]. For convenience, the term adult plant resistance (APR) is commonly used to describe PR observed in the field [11,12]. Field phenotyping for PR or APR is currently the major bottleneck in breeding for durable resistance as, in most cases, assessment can only be performed annually and is affected by environmental inconsistencies reducing phenotypic resolution. Furthermore, PR at the seedling stage can only be analyzed using a combination of uniform inoculation (use of settling tower) and quantitative manual assessments of traits, including infection frequency and delayed latency period, and therefore goes undetected using non-quantitative inoculation and phenotyping methods [13].

Visible macroscopic infection phenotypes provide an intuitive estimation of the disease resistance that has been traditionally utilized by breeders and researchers with experience in analyzing field trial data. However, the infection ratings typically represent the end point of the plant–pathogen interactions and deliver no information about the early and intermediate steps and involved resistance mechanisms. This approach also often combines resistance responses with entirely different backgrounds, thus dramatically lowering the sensitivity and resolution of genome-wide association scans (GWAS).

Pathogen invasion is usually associated with rapid and dramatic gene expression, metabolism, and biochemistry changes [14–16]. Therefore, splitting the observation of the plant–pathogen interactions into a time series, starting from very early time points, may provide valuable information on the infection process and plant responses, especially when combined with different data sources, like transcriptomics and metabolomics.

The enormous progress in digital image analysis achieved over the last few decades has enabled a new level of phenotyping based on processing large amounts of complex image data. Furthermore, artificial intelligence methods, such as machine learning (ML) and deep learning (DL), have been developed to focus on optimizing algorithms that can learn from data [17,18]. In the context of computer vision, these algorithms are used to enable computers to extract, analyze, and understand visual data. A key difference between ML and DL is the human input required to train the algorithms. ML algorithms are typically trained using a large amount of labeled data with a manual selection of features, where the correct output for a given input is known in advance. This allows the algorithm to learn to make predictions based on the patterns it finds in the image data. However, handcrafted-based ML models strongly depend on selecting descriptive and robust features.

In contrast, DL algorithms can learn more autonomously by building multiple layers of interconnected neurons that can learn to extract and analyze complex patterns in data without manual selection of the features. This allows them to make more accurate predictions and handle more complex visual data than traditional ML algorithms. Convolutional neural networks (CNNs) are a class of deep neural networks commonly applied to analyze images. A typical CNN has a multilayer architecture with input and output layers and several hidden layers, which are important for learning specific abstract features. A neuronal network needs a large amount of training data to achieve high accuracy, but it typically will outperform ML-based models [19,20]. For example, in [21], using a dataset of about 10,000 microscopy images of each class, the CNN models outperformed the classical ML models by about 10% accuracy, achieving a 98% true-positive rate and below 3% false-positive rate of fungal structure prediction.

Measuring visible infection symptoms using digital methods (macrophenotyping) resembles the traditional field-based disease rating but with higher accuracy, which makes this method attractive to breeders. In addition, phenotypic assessments of foliar diseases can be precisely measured using a detached leaf assay under controlled environmental conditions allowing for exact and reproducible quantification of pathogen growth. Several digital methods for phenotyping disease resistance traits have been previously developed that are reviewed by Kuska et al. [22]. However, most focus on a field or greenhouse application or require substantial manual input. Fully automated robotized systems using detached leaf assays are less common but crucial for precise phenotyping of large sets of genotypes, such as gene bank collections or large breeding populations. Here, we discuss the opportunity to apply a high-throughput digital phenotyping platform to precisely identify PR to cereal foliar diseases at early growth stages for improved disease control outcomes. We also investigate approaches to combine these technologies with newly developed enabling genomic tools to enhance breeding efficiency and the durability of disease resistance in cereals.

Recently, digital phenotyping systems for macroscopic and microscopic phenotyping were developed and combined in a platform for deep characterization of cereal–mildew interactions [21,23]. The macrophenotyping module of the system was newly utilized to accurately phenotype a large collection of 8,316 German winter wheats derived from the German Federal Gene bank to perform GWAS study on powdery mildew resistance. The study identified 51 marker–trait associations (MTAs), where 11 of them in chromosomal regions are previously not associated with powdery mildew resistance. Further examination of the candidate genes underlying the respective MTAs using the most relevant winter wheat reference genome/s identified gene classes not previously associated with resistance to biotrophic pathogens. Despite the presence of population-specific MTAs, this study highlights the efficacy of the approach to identify resistance alleles with diverse mechanisms of action [24].

**Fig. 2.**
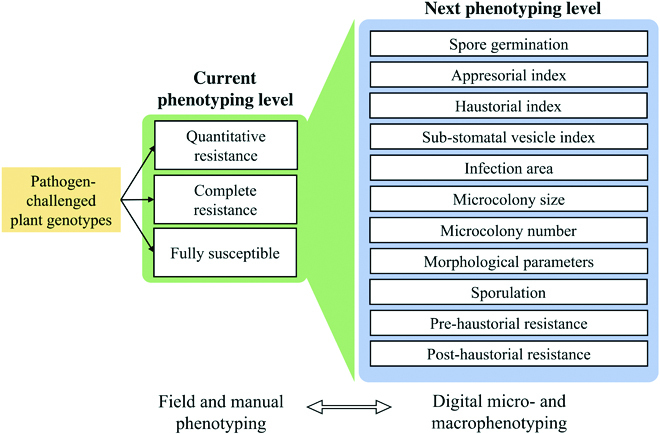
The current and next level of resistance phenotyping for diseases caused by biotrophic fungal pathogens. Currently, phenotyping is based on field and greenhouse trials with operator-specific manual input. High-throughput digital micro- and macroimaging enable the next phenotyping level and more nuanced dissection of the mechanisms of the resistance response. Both levels can communicate and complement each other, combining their benefits such as high-throughput phenotyping of field trials and macroscopic accuracy.

Digital macrophenotyping in detached leaves is an important transition from the field to a fully controlled environment. However, microscopic observations are the key to the next level of disease phenotyping (Fig. 2) [21,25]. They allow much closer insights into the resistance mechanisms, thus significantly increasing the resolution of the MTA studies.

The BluVision microphenotyping platform previously described by Lück and Douchkov [21] can split the quantitative and qualitative powdery mildew resistance phenotypes into microscopic parameters, such as haustoria formation, microcolony counts, and size, and derive phenotypes such as pre- or posthaustorial defense and colony growth rate, helping to efficiently identify and characterize diverse PR mechanisms in cereal crops.

In addition, modules for microphenotyping cereal–rust interactions are in development. Its capabilities have recently been extended using new software modules to phenotype wheat stripe rust (*Puccinia striiformis* f. sp. *tritici*) and wheat leaf rust (*P. triticina*). The next logical step for this technology is to apply it to phenotype PR to agronomically important necrotrophic pathosystems such as Septoria tritici blotch (*Zymoseptoria tritici*), barley net blotch (*Pyrenophora teres*), *Ramularia collo-cygni*, and scald (*Rhynchosporium commune*) diseases. There is also the expectation that microphenomics platforms could be optimized for broad-leaved crops such as legumes and medicinal and horticultural crop species.

Digital phenotyping at the seedling stage in cereals is also applicable for nonhost interactions where, in some instances, variation in cryptic infection (only microscopic symptoms) can be accurately quantified and used to phenotype the variation explained by the underlying genes. The methodology was recently validated by remapping the major effect of quantitative trait locus (QTL) on the long arm of chromosome 5H in barley for nonhost resistance to the wheat-adapted powdery mildew pathogen [21].

The ongoing genomic advancements fueled by reduced sequencing costs and bioinformatic innovation now permit enhanced quantitative genetics, rapid gene isolation, and subsequent functional studies in cereal crops. Most recently, mining diverse landraces, historical cultivars, and ancient relative progenitor species, although not conceptually novel, has been coupled with recent advances in sequencing and marker technologies, facilitating improved capability and power for GWAS [26] and genomic selection [27]. Accurate trait dissection, however, relies on robust quantitative datasets free of operator-specific qualitative rating assessments and human error. The recent transition in resistance breeding away from traditional biparental crosses previously used for QTL analysis and mapping of Mendelian traits to large, diverse germplasm collections used for GWAS highlights the ongoing requirement for improved quantitative phenotyping technologies. The accurate and reproducible quantification of foliar diseases in cereals, such as mildew and rusts delivered by new approaches in digital agriculture such as microphenomics, can provide reliable data for GWAS informing downstream gene cloning and genomic selection applications. Furthermore, the ability to streamline and prioritize PR mechanisms during the phenotypic process provides encouraging potential for enhanced durable resistance in cereals and potentially other crops of interest.
